# The ABA/LANCL1-2 Hormone/Receptors System Controls ROS Production in Cardiomyocytes through ERRα

**DOI:** 10.3390/biomedicines12092071

**Published:** 2024-09-11

**Authors:** Sonia Spinelli, Lucrezia Guida, Mario Passalacqua, Mirko Magnone, Bujar Caushi, Elena Zocchi, Laura Sturla

**Affiliations:** 1 Laboratory of Molecular Nephrology, IRCCS Istituto Giannina Gaslini, Via Gerolamo Gaslini 5, 16147 Genova, Italy; 2 Section of Biochemistry, Department of Experimental Medicine, University of Genova, Viale Benedetto XV 1, 16132 Genova, Italy; l.guida@unige.it (L.G.); mario.passalacqua@unige.it (M.P.); mirko.magnone@unige.it (M.M.); bujar.caushi@edu.unige.it (B.C.); ezocchi@unige.it (E.Z.)

**Keywords:** ROS-producing enzymes, ROS-scavenging enzymes, mitochondrial proton gradient, COX2, XO, NOX4, SOD2, GPX4, ERRα

## Abstract

Rat H9c2 cardiomyocytes overexpressing the abscisic acid (ABA) hormone receptors LANCL1 and LANCL2 have an increased mitochondrial proton gradient, respiration, and vitality after hypoxia/reoxygenation. Our aim was to investigate the role of the ABA/LANCL1-2 system in ROS turnover in H9c2 cells. H9c2 cells were retrovirally infected to induce the overexpression or silencing of LANCL1 and LANCL2, without or with the concomitant silencing of the transcription factor ERRα. Enzymes involved in radical production or scavenging were studied by qRT-PCR and Western blot. The mitochondrial proton gradient and ROS were measured with specific fluorescent probes. ROS-generating enzymes decreased, ROS-scavenging enzymes increased, and mitochondrial ROS were reduced in LANCL1/2-overexpressing vs. control cells infected with the empty vector, while the opposite occurred in LANCL1/2-silenced cells. The knockdown of ERRα abrogated all beneficial effects on ROS turnover in LANCL1/2 overexpressing cells. Taken together, these results indicate that the ABA/LANCL1-2 system controls ROS turnover in H9c2 via ERRα. The ABA/LANCL system emerges as a promising target to improve cardiomyocyte mitochondrial function and resilience to oxidative stress.

## 1. Introduction

Abscisic acid (ABA) is a hormone conserved across kingdoms, including modern plants and animals [1]. In mammals, various cell types produce ABA, which participates in a range of tissue-specific physiological functions, such as the control of blood glucose levels, inflammation, cardiomyocyte energy metabolism, neuroprotection, control of adipocyte browning and energy expenditure, and hemopoietic stem cell regeneration [2,3,4,5,6,7,8].

The metabolic functions of ABA are mediated by two receptors, LANCL1 and LANCL2, which are part of the mammalian LANCL protein family that includes three members conserved from bacteria to humans. The ability of LANCL1 and LANCL2 to bind ABA and their role in facilitating the functional responses to ABA are supported by experimental results [9,10]. These receptors are expressed in all tissues, with particularly high levels of LANCL1 found in the brain, and the heart displaying some of the highest LANCL1 expression among non-neurological tissues [10]. ABA regulates the expression of both receptors, and the two receptors LANCL1 and LANCL2 also exhibit reciprocal regulation. In previous studies, we found that LANCL1 expression was significantly higher in the quadriceps muscles of LANCL2 knockout mice compared with wild-type controls. Chronic oral ABA treatment further elevated LANCL1 expression at both the protein and mRNA levels [10]. Similarly, the silencing of LANCL1 induced a marked increase in LANCL2 expression in L6 muscle cells [10]. Both LANCL1 and LANCL2 receptors and chronic ABA treatment modulate the AMPK/PGC-1α/Sirt1 axis and GLUT4 expression in the muscle cells, cardiomyocytes, and skeletal muscles of wild-type mice. These findings, along with the redundancy of ABA receptors, underscore the physiological importance of the ABA/LANCL hormone/receptor system in mammals.

Recent studies have highlighted the significant role of ABA and its mammalian receptors, LANCL1 and LANCL2, in cardiomyocytes’ adaptive response to hypoxia [6]. Moreover, in LANCL1/2-overexpressing H9c2 cells compared with LANCL1/2-silenced cells we observed an improved cell vitality and mitochondrial function after hypoxia/reoxygenation. This improvement was linked to increased nitric oxide (NO) production and resulted in a higher mitochondrial proton gradient (ΔΨ), mitochondrial DNA content, and respiration. These effects were mediated through a signaling pathway involving AMPK and PGC-1α. The overexpression or silencing of LANCL1/2 led to significant increases or decreases, respectively, in the transcription, expression, and phosphorylation of AMPK, Akt, and eNOS, as well as in the transcription of NAMPT and Sirt1 [6]. Additionally, NADPH levels and the NADPH/NADP ratio were significantly elevated in the overexpressing H9c2 cells compared to the silenced ones. NADPH plays a crucial role in NO synthesis and is essential for maintaining cellular redox balance and antioxidant defense.

In light of this evidence, in this study we studied the role of ABA/LANCL1-2 system in reactive oxygen species (ROS) metabolism in LANCL1/2-overexpressing H9c2 cells compared with LANCL1/2-silenced rat cardiomyocyte H9c2 cells.

Reactive oxygen species (ROS) such as the superoxide anion (O_2_^•−^), hydroxyl radical (^•^OH), and hydrogen peroxide (H_2_O_2_) are produced within cells through endogenous processes, including mitochondrial oxidative phosphorylation and the activity of various enzyme systems, including cyclooxygenase-2 (COX2), xanthine oxidase (XO), and NADPH oxidase (NOX), or they may derive from exogenous sources like ionizing radiation and bacterial infection [11,12]. ROS can be beneficial as subcellular signaling molecules in gene regulatory and signal transduction pathways, but also harmful to cells when ROS levels become unregulated in response to physiological and pathological conditions; in fact, free radicals can adversely affect various important classes of biological molecules, such as nucleic acids, lipids, and proteins, thereby altering the normal redox status and leading to increased oxidative stress. Oxidative stress induced by free radicals has been reported to be involved in several disease conditions, including obesity, diabetes mellitus and metabolic disorders, cardiovascular and respiratory diseases, heart failure, neurodegenerative disorders, rheumatoid arthritis, and in various cancers [13,14].

Cells have evolved multiple mechanisms to defend themselves against oxidative stress by neutralizing ROS. These protective strategies fall into two major categories: non-enzymatic and enzymatic mechanisms. The non-enzymatic approach includes small antioxidant molecules such as vitamin E, vitamin C, β-carotene, glutathione (GSH), coenzyme Q, and bilirubin, which work to neutralize ROS. The second line of defense includes enzymes that detoxify ROS, including superoxide dismutase family (SOD), the seleno-enzyme GSH peroxidase (GPX), and catalase [15].

The regulation of metabolic genes at the transcriptional level plays a vital role in maintaining ROS balance. Gaining deeper insight into how specific transcription factors control ROS metabolism could lead to the development of novel therapeutic approaches. In particular, the estrogen-related receptors (ERRs), transcription factors belonging to the nuclear receptor superfamily are master regulators of cellular energy metabolism and, most recently, are related to ROS metabolism [16,17,18,19]. The ERR subfamily belongs to the larger nuclear receptor superfamily and includes three members: ERRα, ERRβ, and ERRγ [16]. Nuclear receptors, in general, are transcription factors regulated by ligands, which are often small lipophilic hormones, vitamins, or metabolites. These receptors serve as a direct connection between external signals and the regulation of gene expression. ERRα, defined as an orphan receptor as its natural activating ligand is not known, is ubiquitously expressed and the most abundantly expressed isoform of its family and it is involved in the regulation of mitochondrial function and thermogenesis [20]. Moreover, ERRα regulates the transcription of all enzymes that constitute the TCA cycle and a significant number of target genes that code for proteins involved in oxidative phosphorylation (complex I, II, III, IV, V, coenzyme Q, and cytochrome c) [18] and the PGC-1α/ERRα complex regulates the transcription of ROS metabolism targeting genes, encoding several enzymes involved in ROS metabolism, such as NOX4, NOX5, XDH, CAT, GPX, and SOD2 [16,21,22,23].

We observed an impressive (20-fold) increase in ERRα mRNA levels in LANCL1/2-overexpressing, ABA-treated human differentiated adipocytes, both white and brown, in parallel with an increased mitochondrial function; conversely ERRα expression was significantly decreased in LANCL1-2 silenced white and brown adipocytes, suggesting a correlation between the ABA-LANCL1/2 system and ERRα [7]. Moreover, the ABA/LANCL1-2 system activates the AMPK/PGC-1α axis in skeletal and heart muscle, as well as in adipocytes, increasing the expression and also the phosphorylation of both proteins [6,7,10,24], and indeed PGC-1α is a potent coactivator of ERRα [22].

**Figure 1:**
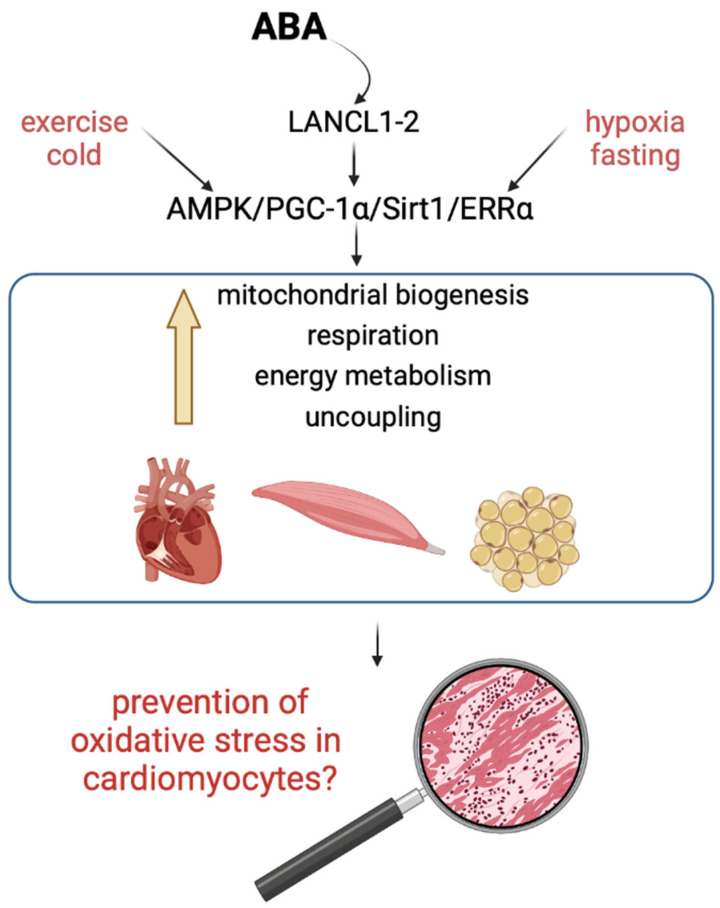
Signaling pathways and functions of ABA in cardiac and skeletal muscle and adipose tissue. The ABA/LANCL1-2 hormone/receptor system, by activating the AMPK/PGC-1α/Sirt1 axis and the orphan-receptor/transcription factor ERRα, stimulates several key mitochondrial functions, such as mitochondrial biogenesis, respiration, and oxidative phosphorylation uncoupling, leading to increased energy availability. Various hormonal/stress signals, including ABA, hypoxia, fasting, exercise, and cold, activate this signaling pathway under physiological conditions. We hypothesized a role for the ABA/LANCL signaling axis in protection against oxygen radicals, a by-product of intense mitochondrial activity.

We recently examined how the ABA/LANCL system influences mitochondrial oxidative metabolism and structural proteins. Overexpressing LANCL1/2 significantly boosted mitochondrial quantity, the activity of OXPHOS complex I, the proton gradient, and respiration dependent on glucose and palmitate. It also enhanced the transcription of uncoupling proteins and the expression of proteins involved in cytoskeletal, contractile, and electrical functions. Conversely, silencing LANCL1/2 had the opposite effects [25]. These effects are mediated by the transcription factor ERRα, which acts upstream of the AMPK/PGC1-α pathway and is regulated by the ABA-LANCL1/2 system [25]. The ABA/LANCL1-2 hormone/receptor system emerges as a new controller of cardiomyocyte “fitness” improving cardiac function and resilience to hypoxic and dysmetabolic conditions via a reciprocal transcriptional stimulation with ERRα (Figure 1) [25,26].

The aims of this study were (i) to investigate the role of the ABA/LANCL1-2 system in ROS production and detoxification by comparing the expression levels of the principal enzymes involved in these pathways and ROS content in LANCL1/2-overexpressing vs. LANCL1/2-silenced rat H9c2 cardiomyocytes; (ii) to investigate a possible role for ERRα in the ABA/LANCL1-2 signaling pathway in LANCL1/2-overexpressing H9c2 cells. The choice of H9c2 cells as the cell experimental model was motivated by the previous observation that the overexpression of LANCL1/2 protects these cells from hypoxia/reoxygenation-induced damage, a consequence of ROS generation [6]. 

## 2. Materials and Methods

### 2.1. Cell Culture

H9c2 rat cardiomyocytes were purchased from ATCC (LGC Standards s.r.l. Milan, Italy). Cells were incubated in high-glucose DMEM (Sigma-Aldrich, Milan, Italy) containing 10% heat-inactivated fetal bovine serum (Sigma-Aldrich, Milan, Italy) and 1% penicillin–streptomycin (Sigma-Aldrich, Milan, Italy) at 37 °C in a humidified atmosphere with 5% CO_2_. The cells were subcultured upon reaching 70–80% confluence. Experiments were performed using cells between Passage 5 and 8.

### 2.2. Lentiviral and Retroviral Cell Transduction

The lentiviral plasmids for silencing rat LANCL1 (SHL1), LANCL2 (SHL2), and ERRα (SHERRα) (plasmid ID: VB010000-0005mme, VB181016-1107sen, VB181016-1124zjp, VB221005-1073jxq), and the respective control vectors (SCR for a control scramble shRNA), were purchased from Vector Builder (Chicago, IL, USA). Lentiviral transductions were performed as described in [6,27]. Briefly, HEK293T cells were plated at a density of 6 × 10^5^ cells per 6 cm plate in Dulbecco’s modified Eagle medium supplemented with 10% fetal bovine serum and 0.1% penicillin–streptomycin. After 24 h, when the cells reached 60% to 70% confluency, they were cotransfected with 4 µg of the vector of interest and Lentiviral Packaging Mix using TransIT-293 Transfection Reagent (TEMA Ricerca, Bologna, Italy). Twelve hours later, the medium was replaced with Dulbecco’s modified Eagle medium containing 20% fetal bovine serum and 10% penicillin–streptomycin to enhance viral production. The supernatant containing lentiviral particles was collected 24 and 48 h post-transfection, filtered through a 0.45 µm filter, and used to infect 2.5 × 10^6^ H9c2 cells in the presence of protamine sulfate (final concentration 1 mg/mL). After the second cycle of infection, cells were selected using puromycin at a concentration of 4 µg/mL. Transduction efficiency was estimated 72 h post-selection using qRT-PCR and Western blot analyses. hLANCL1 (OVL1) and hLANCL2 (OVL2) were overexpressed in rat H9c2 cardiomyocytes with pBABE vectors, constructed as described in [10], with the empty vector pBABE (Addgene, Watertown, MA, USA) as negative control (PLV). For retroviral transduction, we followed the same protocol as described above for lentiviral transduction, using HEK 293 Phoenix cells instead of 293T.

### 2.3. Cell Viability Assay

H9c2 viability was determined using the resazurin assay as previously described [28]. Briefly, exponentially growing cells were seeded in 96-well microplates at a density of 10 × 10^3^ cells/well and incubated in complete DMEM medium overnight. After 24 h, cells were treated for 3 h with two different concentrations of H_2_O_2_ (200 µM and 600 µM, respectively) in 100 µL of serum free DMEM medium. After H_2_O_2_ treatment, 20 µL of resazurin at a concentration of 0.15 mg/mL in PBS was added to each of the well and then incubated at 37 °C for another 4 h. Next, the absorbance signal was measured at 570/590 nm (excitation/emission wavelengths) using a plate reader (CLARIOstar plus; BMG Labtech Ortenberg, Germany). The results are shown as the percentage of surviving cells compared with the total 100% of untreated cells.

### 2.4. Lipid Peroxides Measurement

Lipid peroxidation was determined using the fluorescence probe C11-BODIPY 581/591 (Life Technologies, Milan, Italy) according to the manufacturer’s instructions. In the presence of lipid hydroperoxides, C11-BODIPY 581/591 shifts fluorescence emission peak from 590 nm (red) to 510 nm (green) of the phenylbutadiene segment of the fluorophore [29], resulting in a lower 590/510 ratio compared to untreated cells. Briefly, cells were plated and treated with H_2_O_2_ (200 µM and 600 µM, respectively) as described in Section 2.3. After H_2_O_2_ treatment, C11-BODIPY 581/591 reagent was added to each well with a final concentration of 1 µM and incubated for 30 min. After incubation, the medium was removed and the cells were washed three times with PBS and then the fluorescence emission at 590 and 510 nm was determined using Clariostar plus plate reader. 

### 2.5. qRT-PCR Analysis

After 18 h starvation, incubating cells in low-glucose DMEM without serum, cardiomyocytes were treated or not with 100 nM ABA for 4 h and subjected to subsequent analyses. RNA extraction, cDNA synthesis, and quantitative real-time PCR (qRT-PCR) were performed as described previously [7]. Gene-specific primers were purchased from Sigma-Aldrich (Milan, Italy) and are listed in Table 1. Comparisons in the gene expression were performed using the iQ5 Optical System Software version 1.0 (Bio-Rad Laboratories, Milan, Italy) by the 2^−△△Ct^ method [7]. Hprt1 was used as the housekeeping gene for normalization.

### 2.6. Western Blot Analysis

After transduction, 1 × 10^6^/well H9c2 cells were plated in 6-well plates and allowed to adhere for 24 h. After serum deprivation and low-glucose DMEM incubation for 18 h, cardiomyocytes were washed once with HBSS and then incubated for 1 h at 37 °C with or without 100 nM ABA. Thereafter, cells were lysed in ice-cold lysis buffer [20 mM Tris-HCl (pH 7.4), 150 mM NaCl, 1 mM EDTA, 1% NP40 and Protease Inhibitor Cocktail] and briefly sonicated, and the protein concentration was determined according to a standard Bradford assay. Proteins (30 μg) were separated by SDS-PAGE and transferred to nitrocellulose membranes (Bio-Rad, Milan, Italy). Membranes were blocked for 1 h with TBST containing 5% non-fat dry milk and incubated for 1 h at room temperature with primary antibodies (Table 2). Following incubation with the appropriate secondary antibodies (Table 2), band intensities were quantified by the Quantity One SW software Version 4.1 (Bio-Rad, Milan, Italy) using standard ECL (GE Healthcare, Milan, Italy).

### 2.7. ROS Detection Assays

Two different fluorescent ROS-sensitive probes were used, dichlorodihydrofluorescein diacetate (H2DCFDA) and MitoSOX Red [31,32]. DCFDA diffuses into the cells and is then deacetylated by cellular esterases to a non-fluorescent compound, which becomes fluorescent upon oxidation by ROS, thus behaving as a “whole cell” sensor of ROS production. MitoSOX Red is instead specifically targeted to mitochondria and allows the detection of mitochondrial ROS.

For DCFDA-based assays, H9c2 cells were cultured overnight at 1 × 10^4^/well in a 96-well plate in high-glucose DMEM with 10% FBS and 1% penicillin–streptomycin, then washed once with HBSS and loaded for 45 min with 10 μM H2DCFDA at 37 °C in HBSS without or with 100 nM ABA. At the end of the incubation, the supernatant was removed, cells were washed once with HBSS, and 100 μL HBSS was added to each well. The fluorescence (excitation at 488 nm and emission at 530 nm), monitored with a plate reader (Clariostar plus; BMG Lab Technologies), was calculated by the mean fluorescence from 5 acquisitions/well; each experimental condition was assayed in at least 8 wells.

For MitoSOX-based assays, MitoSOX Red reagent (5 mM) (Thermo Fisher Scientific, Waltham, MA, USA) was resuspended in DMSO and diluted in HBSS to obtain a final concentration of 5 μM as a working solution. Cells were incubated in 500 μL of working solution for 20 min at 37 °C in a CO_2_ incubator and positive staining was evaluated by a Leica TCS SP2 confocal microscope equipped with a x60 N.A. 1.4 oil immersion objective. Fluorescence intensity was analyzed after background subtraction with FIJI ImageJ software (version 2.14.0/1.54f), using quantitative analysis based on the intensity measurement of specific selected ROIs.

### 2.8. Statistical Analysis

All data were analyzed with the GraphPad Prism software version 7 (GraphPad Software, San Diego, CA, USA). All parameters were tested by two-tailed Student’s *t*-test. *p*-values less than 0.05 were considered significant.

## 3. Results

### 3.1. Overexpression and Silencing of LANCL1 or LANCL2 in H9c2 Cells

**Figure 2:**
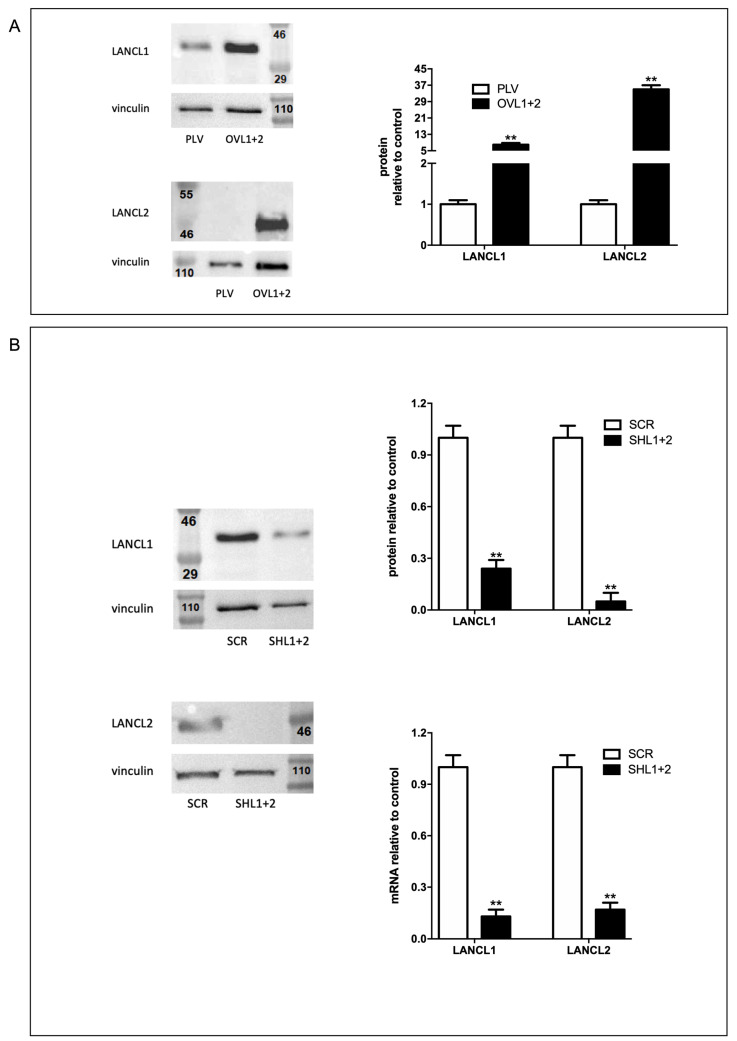
Overexpression and silencing of LANCL1/2 in H9c2 rat cardiomyocytes. LANCL1 and LANCL2 were overexpressed (**A**) or silenced (**B**) in H9c2 cells by lentiviral infection. (**A**) Left panel, representative Western blots of LANCL1 and LANCL2 protein expression in cells overexpressing both LANCL proteins (OVL1+2) or infected with the empty vector (PLV); right panel, densitometric quantitation of the LANCL proteins expression in the same cell types normalized on PLV control and relative to vinculin. Values are normalized against vinculin, as a housekeeping protein. (**B**) Left panel, representative Western blots of LANCL1 and LANCL2 in cells silenced for the expression of both proteins (SHL1+2) or infected with the scrambled sequences (SCR); upper right panel, densitometric quantitation of the LANCL proteins in the same cell types, normalized on SCR control and relative to vinculin; lower right panel, LANCL1/2 mRNA levels relative to control in LANCL1/2-silenced cells. Values are normalized against vinculin, as a housekeeping protein. The exposure times of the Western blots shown in (**A**,**B**) were different (30 s for (**A**) and 180 s for (**B**)), in order to visualize the much lower protein levels in LANCL1/2-silenced H9c2 cells. Data are the mean ± SD from at least three experiments. ** *p* < 0.001 relative to control cells (PLV for overexpression or SCR for silencing) by unpaired Student’s *t*-test.

In order to study the functions of ABA and its receptors on the control of oxidative stress and ROS metabolism in cardiomyocytes, H9c2 cells were transfected to obtain double-silenced or overexpressing cells for both receptors LANCL1 and LANCL2. The overexpression (OVL1+2) or silencing (SHL1+2) of LANCL1/2 was conducted using retroviral or lentiviral infection, respectively, and confirmed by Western blot. The fold-increase in protein expression was approximately 6 for LANCL1 and 30 for LANCL2 (Figure 2A) compared with control cells infected with the empty vector (PLV). Moreover, in silenced cells we observed a reduction in the transcription and expression of both receptors, analyzed by qRT-PCR and Western blot, 76% for LANCL1 and 95% for LANCL2 (Figure 2B).

### 3.2. Overexpression of LANCL1 and LANCL2 Protects H9c2 Cardiomyocytes from H_2_O_2_ Induced-Oxidative Stress

**Figure 3:**
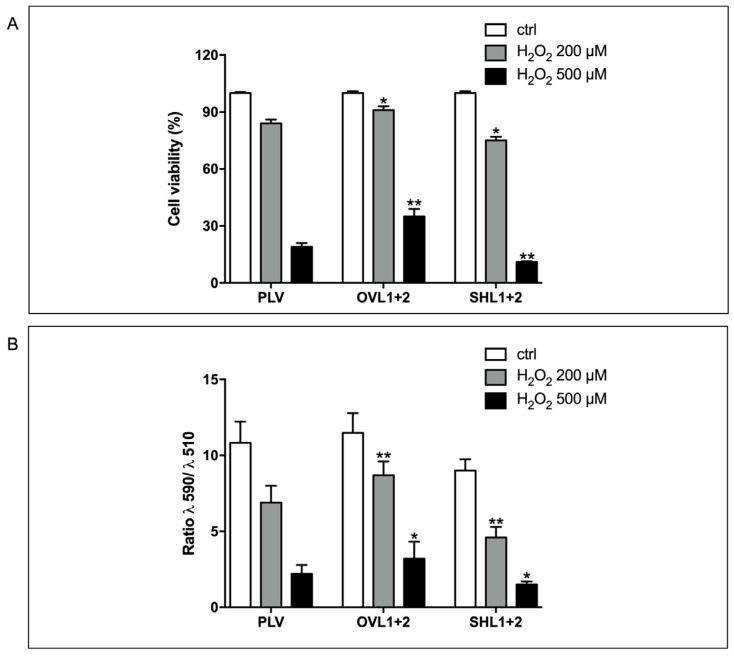
Overexpression of LANCL1 and LANCL2 protects H9c2 cardiomyocytes from H_2_O_2_ induced-oxidative stress. H9c2 overexpressing LANCL1 and LANCL2 (OVL1+2), double-silenced (SHL1+2), and control cells (PLV) were incubated in the absence or in the presence of 200 or 600 µM H_2_O_2_ for 3 h. (**A**) The cell viability was determined by resazurin. Results are expressed as the percentage of cell survival relative to untreated cells. (**B**) Intracellular lipid hydroperoxides production was detected with the fluorescent probe C11-BODIPY 581/591. Results are expressed as the fluorescence intensity ratio at 590/510 nm. Histograms summarize the quantitative data of the mean ± SD of three independent experiments. * *p* < 0.01, ** *p* < 0.001 relative to the respective control cells by unpaired *t*-test.

To induce oxidative stress in H9c2 cardiomyocytes, we used H_2_O_2_, which is commonly used to evoke cellular oxidative damage [33]. H9c2 cardiomyoblasts overexpressing and silenced for both LANCL1 and LANCL2 and in parallel control PLV cells were treated with H_2_O_2_ to elucidate the protective effects of the LANCL1/2 system; after inducing oxidative stress, the viability of cells and lipid peroxidation were determined. The cell viability of cells treated with 600 µM H_2_O_2_ was significantly reduced in control and silenced cells, with a 11% survival rate, compared with that of overexpressing LANCL1/2 cells (35%) (Figure 3A). Silenced cells were also more sensitive to oxidative stress after treatment of the cells with the lowest concentration of H_2_O_2_ (66% survival rate compared with 81% and 92% obtained in control and overexpressing cells, respectively).

To determine the levels of H_2_O_2_-induced lipid peroxidation, H9c2 cells (LANCL1/2-overexpressing vs. double-silenced and control) were incubated with C11-BODIPY 581/591, a sensitive fluorescent reporter for lipid hydroperoxides. Channel emission signal ratios at 590 nm and 510 nm were used to quantify cell lipid peroxidation. In control cells, the 590/510 ratio was high, whereas when the cells were treated with H_2_O_2_ the ratios were lower. In LANCL1/2 overexpressing cells, the amount of lipid peroxides produced after H_2_O_2_ treatment at both concentrations was about half that of control and silenced cells: the ratios were 8.7, 4.6, and 6.9 after treatment with 200 µM H_2_O_2_ and 3.2, 1.1, and 2.2 after treatment with 600 µM H_2_O_2_ in overexpressing, silenced, and control cells, respectively (Figure 3B). Interestingly, untreated LANCL1/2-silenced cells show a higher basal amount of lipid hydroperoxides compared with overexpressing and control cells. These results suggested that LANCL1/2 expression levels play an important role in the control of oxidative stress and prompted us to investigate the expression of radicals-generating and -scavenging enzymes related to oxidative stress.

### 3.3. Overexpression of LANCL1 and LANCL2 Decreases, While Their Combined Silencing Increases, the Transcription and Expression of Radicals-Generating COX2, XO, and NOX4

**Figure 4:**
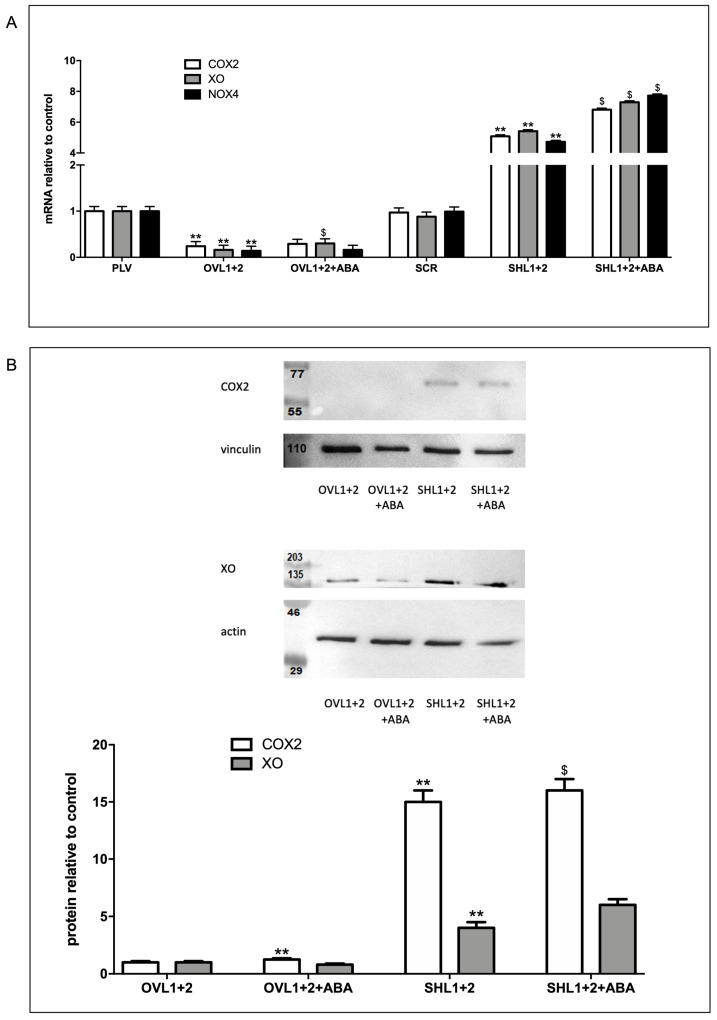
Radicals-generating enzymes are reduced in LANCL1/2-overexpressing vs. double-silenced H9c2 cells. (**A**) qRT-PCR analysis of the transcription of radicals-generating enzymes (COX2, XO, and NOX4) in cells overexpressing (OVL1+2) or silenced (SHL1+2) for LANCL1 and LANCL2 proteins and incubated in the absence or in the presence of 100 nM ABA for 4 h. Results are expressed relative to control ABA-untreated, PLV cells. ** *p* < 0.001 relative to untreated control cells (PLV or SCR) and $ *p* < 0.02 relative to ABA-untreated OVL1+2 or SHL1+2 cells by unpaired *t*-test. (**B**) Upper panel, representative Western blot image of COX2 and XO proteins in LANCL1/2-overexpressing or silenced cells, treated or not with 100 nM ABA for 4 h. Lower panel, histograms are the mean ± SD from at least three experiments. Results are expressed relative to ABA-untreated OVL1+2 cells. Values are normalized against vinculin, as a housekeeping protein. Data are ** *p* < 0.001 relative to untreated OVL1+2 cells and $ *p* < 0.02 relative to ABA-untreated SHL1+2 cells by unpaired *t*-test.

In order to understand the role of the LANCL proteins in inflammation and ROS production in H9c2, the expression levels of radicals-generating COX2, XO, and NOX4 were evaluated in cells overexpressing or silenced for both LANCL1 and LANCL2, treated or not with ABA, using qRT-PCR and Western blot analysis. In LANCL1/2-overexpressing H9c2 cells (OVL1+2), the mRNA levels for COX2, XO, and NOX4 were all significantly reduced (by 70-80%) compared with controls infected with the empty vector PLV; conversely, in double-silenced cells (SHL1+2), mRNA levels for the same enzymes increased approx. 6-fold over control cells infected with the scrambled sequences (SCR) (Figure 4A). Similar results were observed at the protein level for COX2 and XO, as measured by Western blot (Figure 4B). These results indicate that LANCL1/2 expression levels inversely control the transcription and expression of COX2, XO, and NOX4, with low levels of LANCL1/2 expression favoring an inflammatory and carbon and oxygen radicals-producing cell phenotype.

### 3.4. LANCL1/2-Overexpression Increases, While Their Double-Silencing Decreases, the Transcription and Expression of the Radical Scavenging Enzymes SOD2 and GPX4

**Figure 5:**
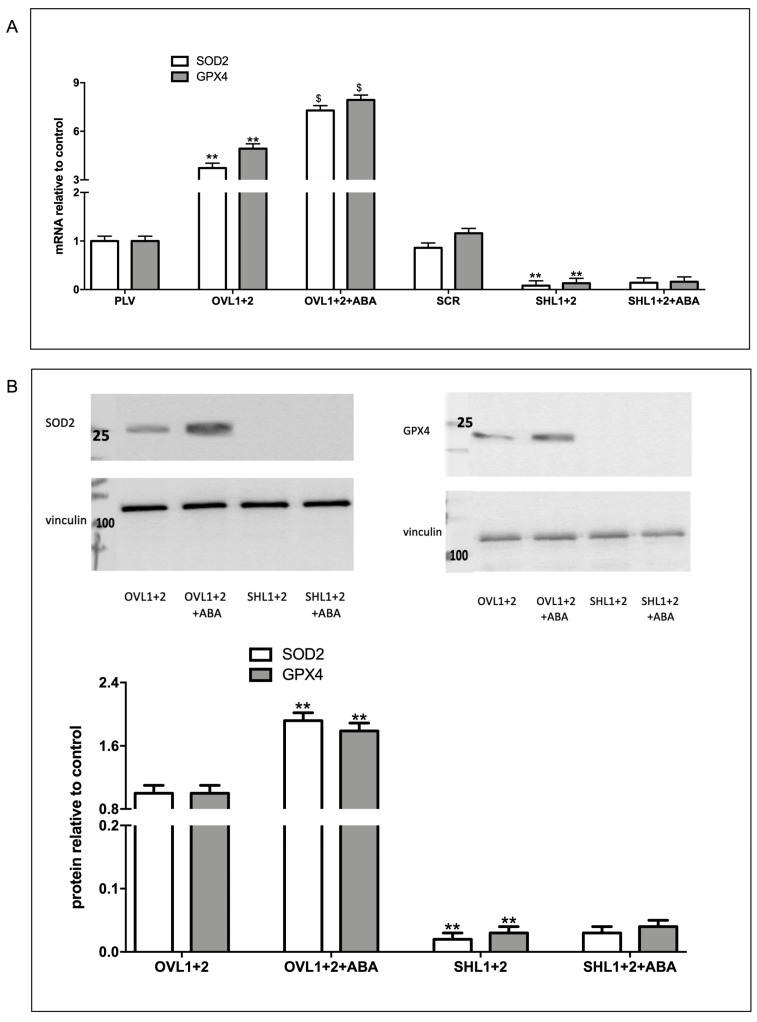
Radicals-scavenging enzymes are increased in LANCL1/2-overexpressing vs. double-silenced H9c2 cells. (**A**) qRT-PCR analysis of the transcription of radicals-scavenging enzymes (SOD2 and GPX4) in cells overexpressing (OVL1+2) or silenced (SHL1+2) for LANCL1 and LANCL2 and incubated in the absence or in the presence of 100 nM ABA for 4 h. Results are expressed relative to control ABA-untreated PLV cells. ** *p* <0.001 relative to untreated control cells (PLV or SCR) and $ *p* < 0.02 relative to ABA-untreated OVL1+2 or SHL1+2 cells by unpaired *t*-test. (**B**) Upper panel, a representative Western blot image of SOD2 and GPX4 in LANCL1/2-overexpressing or silenced cells, treated or not with 100 nM ABA for 4 h. Lower panel, histograms are the mean ± SD from at least three experiments. Results are expressed relative to ABA-untreated OVL1+2 cells. Values are normalized against vinculin, as a housekeeping protein. Data are ** *p* < 0.001 relative to untreated OVL1+2 cells by unpaired *t*-test.

To further investigate a possible role of the ABA/LANCL1-2 system in ROS metabolism, we analyzed the expression of two important enzymatic antioxidants, superoxide dismutase (SOD2) and glutathione peroxidase (GPX4), which can directly or indirectly catabolize ROS to protect cells. Interestingly, overexpression of LANCL1/2 (OVL1+2) significantly increased the transcription of both SOD2 and GPX4, 4- and 5-fold, respectively, as compared with control cells, infected with the empty vector (PLV) (Figure 5A). Moreover, treatment of the cells with 100 nM ABA further significantly increased the mRNA levels of both SOD2 and GPX4. The expression of target genes identified in LANCL1/2-overexpressing H9c2 cells was also studied in cells where LANCL1/2 were both silenced. The results obtained were the opposite: the mRNA levels of SOD2 and GPX4 were significantly lower in the silenced cells compared to controls and did not increase with ABA treatment (Figure 5A). Western blot analysis confirmed approx. 80-fold higher protein levels of SOD2 and GPX4 in LANCL1/2-overexpressing vs. double-silenced H9c2 cells (Figure 5B). These results suggested that the ABA/LANCL system is involved not only in the control of ROS production but also in antioxidant defense. Recently, the NADPH/NADP ratio (2.1 vs 1.6) in LANCL1/2-overexpressing vs. double-silenced H9c2 cells was determined [6] and it was significantly higher in overexpressing cells. NADPH is required for the activity of glutathione reductase (GR), which keeps GSH in the reduced form, essential for the antioxidant activity of glutathione peroxidase (GPX). These results are in line with the increased expression of GPX4 in overexpressing cells, suggesting that all enzymatic activities involved in the GSH-mediated detoxification system, which include SOD, GPX, and GR, are activated.

### 3.5. LANCL1/2-Overexpression Decreases and Their Combined Silencing Conversely Increases ROS Content in H9c2 Cells

**Figure 6:**
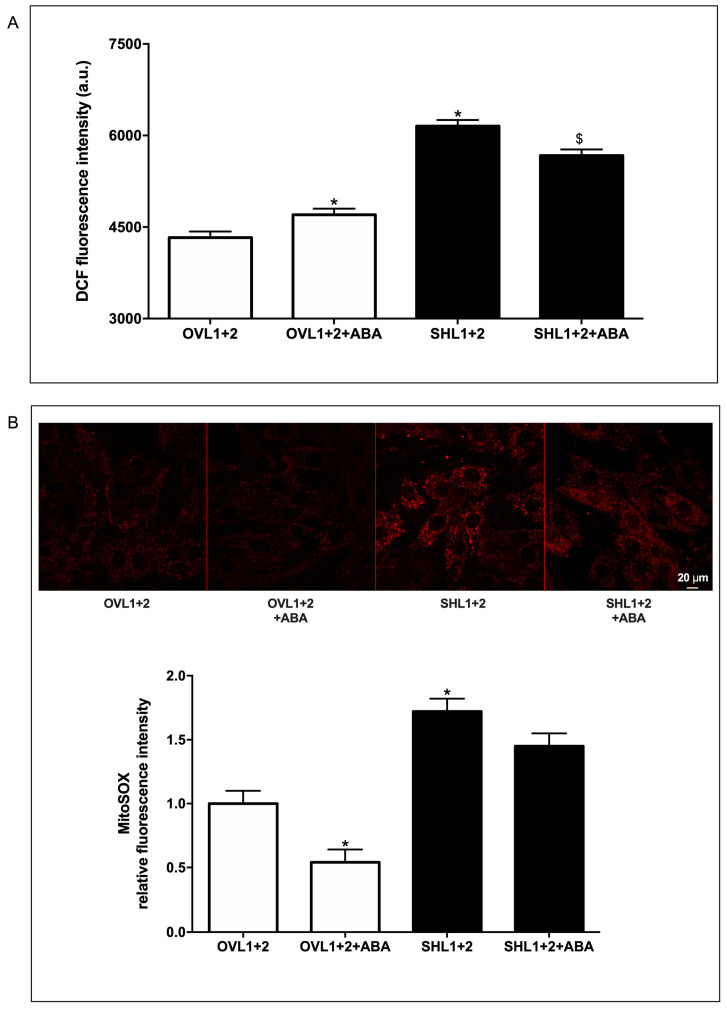
LANCL1/2-overexpressing cells have a reduced ROS content compared with double-silenced H9c2 cells. (**A**) Intracellular ROS production measured by DCF fluorimetric analysis in H9c2 cells. Results are expressed as fluorescence intensity in arbitrary units. (**B**) Mitochondrial superoxide anions were detected by confocal microscopy on MitoSOX-loaded H9c2 overexpressing (OVL1+2) or silenced (SHL1+2) for LANCL1 and LANCL2 proteins and incubated in the absence or in the presence of 100 nM ABA for 4 h. Upper panel, representative images of the cells; lower panel, histograms represent the mean cell fluorescence recorded in three independent experiments, relative to ABA-untreated OVL1+2 cells. * *p* <0.01 relative to untreated OVL1+2 cells and $ *p* < 0.02 relative to ABA-untreated SHL1+2 cells by unpaired *t*-test.

To further confirm the data obtained on gene and protein expression related to ROS metabolism, we analyzed the ROS content with two different methods, using fluorogenic substrates: (i) dichlorodihydrofluorescein diacetate (H2DCFDA), deacetylated by cellular esterases into a non-fluorescent compound, which is subsequently oxidized by ROS into 2′,7′-dichlorofluorescein (DCF) (Figure 6A), and (ii) the Mitochondrial Superoxide Indicator MitoSOX™, a fluorogenic dye specifically targeting mitochondria in live cells, the oxidation of which by mitochondrial superoxide produces a bright red fluorescence (Figure 6B). In both experiments, an increased basal ROS production was observed in LANCL1-2 silenced H9c2 cells compared with overexpressing cells, in agreement with the higher expression of enzymes involved in ROS production (NOX4 and XO) and with the reduction in antioxidant enzymes expression (SOD2 and GPX) observed in double-silenced cells. ABA treatment significantly reduced mitochondrial ROS production in the overexpressing, but not in the double-silenced, cells (Figure 6B).

### 3.6. The ABA/LANCL1-2 System Controls ROS Metabolism via the Transcription Factor ERRα

The results described above indicate that LANCL1/2 proteins control ROS production in H9c2 cells at least in part by upregulating radical-scavenging enzymes and coenzymes and by reducing the activity of ROS-generating enzymes. The estrogen-related receptors (ERRs) are transcription factors belonging to an “orphan receptor” superfamily, which play important roles in the regulation of mitochondrial oxidative metabolism and in the defense against ROS production [14,15,16,17]. In particular, ERRα is a novel redox sensor and effector of a ROS defense program, regulating the transcription of many enzymes involved in ROS metabolism as a significant number of target genes that code for proteins involved in oxidative phosphorylation (complex I–IV, coenzyme Q, and cytochrome c), all of the enzymes that constitute the TCA cycle, NOX4 and NOX5, SOD2, and GPX regulating Sirt3 expression by PGC-1α [16,18,23].

**Figure 7:**
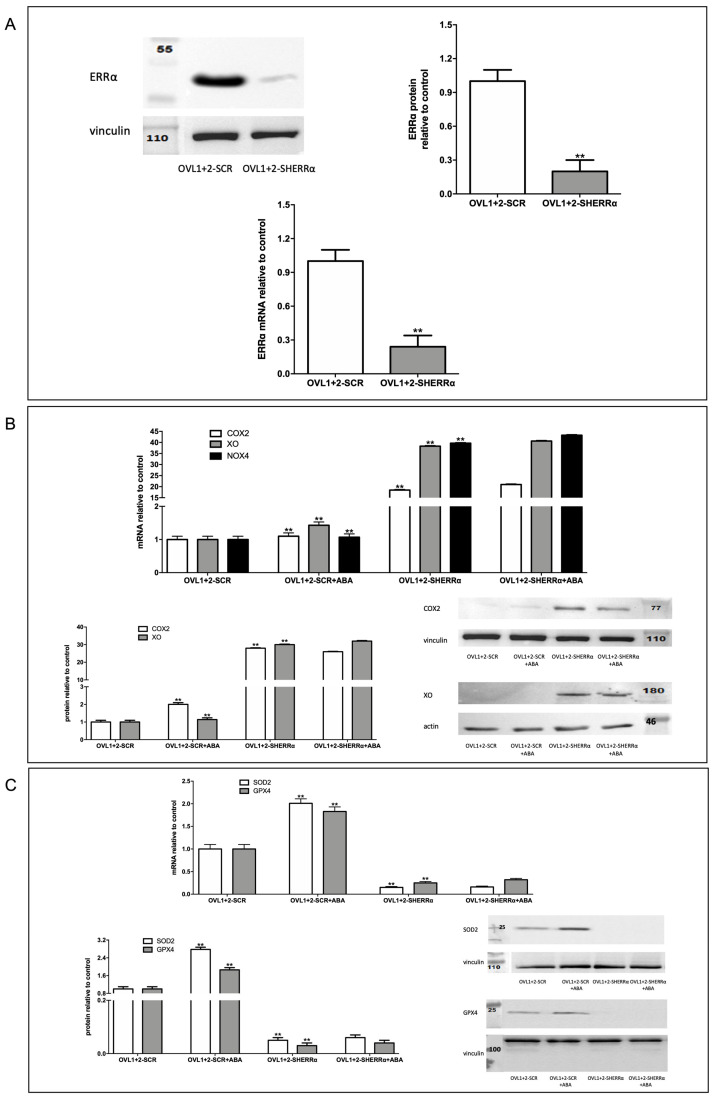
ERRα silencing in LANCL1/2-overexpressing cells increases radicals-generating and decreases radicals-scavenging enzymes. (**A**) OVL1+2 cells were silenced for the expression of ERRα by lentiviral infection. Upper left panel, representative Western blot of ERRα in OVL1+2 cells silenced for the expression of ERRα (OVL1+2-SHERRα) or infected with the empty vector (OVL1+2-SCR); upper right panel, densitometric quantitation of ERRα in the same cell types. Values are normalized against vinculin, as a housekeeping protein; lower panel, ERRα mRNA levels relative to control OVL1+2-SCR cells in ERRα-silenced cells (OVL1+2-SHERRα). ** *p* < 0.001 relative to control OVL1+2-SCR cells by unpaired *t*-test. (**B**) qRT-PCR analysis (upper panel) and Western blot analysis (lower panels) of radicals-generating enzymes (COX2, XO and NOX4) in OVL1+2 cells silenced for ERRα (OVL1+2-SHERRα) and incubated in the absence or in the presence of 100 nM ABA for 4 h. Results are expressed relative to ABA-untreated OVL1+2-SCR cells. ** *p* <0.001 relative to ABA-untreated OVL1+2-SCR cells by unpaired *t*-test. (**C**) qRT-PCR analysis (upper panel) and Western blot analysis (lower panels) of radicals-scavenging enzymes (SOD2 and GPX4) in OVL1+2 cells silenced for ERRα (OVL1+2-SHERRα) and incubated in the absence or in the presence of 100 nM ABA for 4 h. Results are expressed relative to control ABA-untreated OVL1+2-SCR cells. ** *p* <0.001 relative to untreated OVL1+2-SCR cells by unpaired *t*-test.

To investigate the role of ERRα in the protection against ROS production observed in LANCL1/2-overexpressing H9c2, ERRα was silenced by lentiviral infection using a vector containing sequences encoding specific shRNAs. As shown in Figure 7A, the knockdown of ERRα in LANCL1/2-overexpressing cells (OVL1+2-SHERRα) was confirmed by both immunoblot (Figure 7A, upper panels) and qRT-PCR (Figure 7A, lower panel). The expression of ERRα was reduced by approx. 80% relative to control cells infected with the vector containing scrambled silencing sequences (OVL1+2-SCR) (Figure 7A).

To investigate ROS metabolism in LANCL1/2-overexpressing and ERRα-silenced H9c2 cells (OVL1+2-SHERRα), we firstly evaluated the transcription of radicals-generating COX2, NOX4, and XO and of radicals-scavenging SOD2 and GPX4 by qRT-PCR. ERRα silencing in LANCL1/2-overexpressing cells (OVL1+2-SHERRα) significantly increased mRNA levels of all radicals-generating enzymes compared with control cells transfected with the scrambled sequences (OVL1+2-SCR) (Figure 7B). Conversely, transcription of the antioxidant enzymes SOD2 and GPX4 decreased in OVL1+2-SHERRα cells compared with controls (Figure 7C). The increase or decrease in mRNA levels was indeed very marked (logarithmic) as a result of ERRα silencing. Western blot analysis confirmed a significant increase in radicals-generating (Figure 7B, lower panel) and decrease in radicals-scavenging enzymes (Figure 7C, lower panel) in ERRα-silenced LANCL1/2-overexpressing H9c2 cells compared with control cells transfected with the scrambled sequences for ERRα. These results suggested that ERRα is necessary for the decreased expression of radicals-generating enzymes and the increased expression of protecting enzymes observed in LANCL1/2-overexpressing vs. double-silenced cells.

### 3.7. ERRα Silencing Increases ROS Production in LANCL1/2-Overexpressing Cells

**Figure 8:**
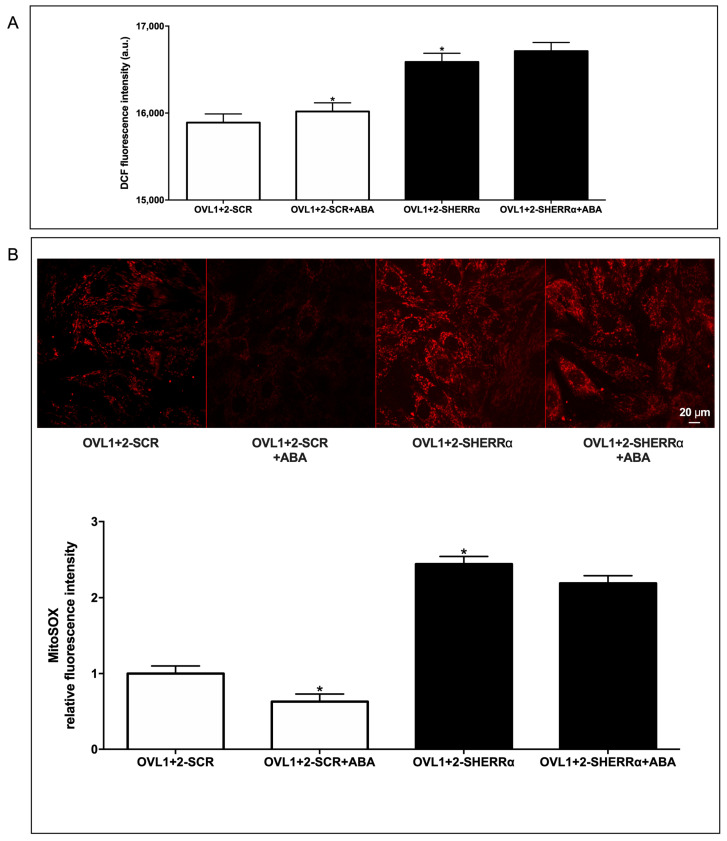
ERRα silencing increases ROS production in LANCL1/2-overexpressing H9c2 cells. (**A**) Intracellular ROS production measured by DCF fluorimetric analysis in H9c2 cells. Results are expressed as fluorescence intensity in arbitrary units with respect to OVL1+2-SCR cells. (**B**) Mitochondrial superoxide anions were detected on MitoSOX-loaded cells by confocal microscopy in H9c2 cells overexpressing LANCL1 and LANCL2 proteins and silenced (OVL1+2-SHERRα) or not (OVL1+2) for ERRα and incubated in the absence or in the presence of 100 nM ABA for 4 h. Upper panel, representative confocal microscopy of the cells; lower panel, histogram summarized quantitative data of the mean ± SD of three independent experiments. * *p* < 0.01 relative to untreated OVL1+2-SCR cells by unpaired *t*-test.

We next investigated the ROS content in ERRα-silenced-LANCL1/2-overexpressing H9c2 cells (OVL1+2-SHERRα) compared with control LANCL1/2-overexpressing cells, transfected with the scrambled sequences for ERRα (OVL1+2-SCR). We quantified ROS using the same methods employed to compare ROS content in LANCL1/2-overexpressing vs. double-silenced cells. ERRα silencing significantly increased ROS production in LANCL1/2-overexpressing cells (OVL1+2-SHERRα) vs. controls (OVL1+2-SCR) as determined with both fluorescent probes (Figure 8A,B), with the highest increase (2.5 times over control values) being observed with the mitochondrial dye MitoSOX™ (Figure 8B). These results indicate that ERRα plays a critical role in the reduced ROS content observed in LANCL1/2-overexpressing vs. double-silenced H9c2 cells (Figure 6). Indeed, a 2.5-fold increase would abrogate the significant reduction in ROS content observed in LANCL1/2-overexpressing vs. double-silenced cells shown in Figure 6. Also noteworthy is the fact that treatment with ABA significantly reduces (by 50%) ROS content in LANCL1/2-overexpressing cells, in spite of its reported stimulation of mitochondrial respiration [6]. Silencing of ERRα abrogates reduction by ABA of mitochondrial ROS production and increases “whole cell” DCFDA fluorescence in LANCL1/2-overexpressing cells.

## 4. Discussion

Collectively, the results obtained in this study outline an unknown regulatory role for the ABA/LANCL1-2 hormone/receptors system in cardiomyocyte protection from ROS-dependent oxidative stress, via the transcription factor ERRα.

A reciprocal relationship emerges between LANCL1/2 expression levels and the transcription and expression of critical ROS-scavenging and ROS-producing enzymes, which depend on the transcription factor ERRα. The result of this regulatory mechanism is that LANCL1/2-overexpressing H9c2 cells have a significantly higher survival rate under a condition of oxidative stress and show a reduced lipid hydroperoxides and mitochondrial ROS content compared with double-silenced cells, in the face of a significantly steeper mitochondrial proton gradient [6]. Thus, LANCL1/2-overexpressing cells appear to be more protected than double-silenced cells against mitochondrial ROS generation, despite having a higher respiratory chain activity.

Several key ROS-producing and -scavenging enzymes appear to be controlled at the transcriptional and translational level by the ABA/LANCL1-2/ERRα system. The radicals-generating enzymes COX2, NOX4, and XO were all significantly reduced in LANCL1-2-overexpressing vs. double-silenced H9c2 cells. COX2, the rate-limiting enzyme in the synthesis of prostaglandins and a key player in inflammation and oxidative stress [34], was reduced 15-fold in LANCL1/2-overexpressing compared with double-silenced cells (Figure 2B). The mRNA levels of NOX4, the most abundant isoform of the H_2_O_2_-generating NADPH oxidases family in cardiomyocytes [35], were 23-fold lower in LANCL1/2-overexpressing vs. double-silenced cells (Figure 2A). Finally, the protein levels of xanthine oxidase (XO), which produces O_2_^−^ after sulfhydryl oxidation as occurs during myocardial ischemia/reperfusion (I/R) [36], were about 6-fold lower in LANCL1/2-overexpressing vs. double-silenced cells (Figure 4B).

Radical-scavenging enzymes were instead increased in LANCL1/2-overexpressing compared with double-silenced H9c2 cells. The transcription and expression of SOD2, a Mn-SOD localized in the mitochondrial matrix, arguably the most important site of cellular ROS production, were 40- and 50-fold higher in LANCL1/2-overexpressing vs. double-silenced cells (Figure 5). The SOD family detoxifies O_2_^−^ through its conversion to H_2_O_2_, which is then reduced to water by GPX, through the oxidation of GSH [37]. GPX4 protein levels were indeed 45-fold higher in LANCL1/2-overexpressing vs. double-silenced cells (Figure 3B). In addition to reducing H_2_O_2_ and small hydroperoxides in general, GPX4 also has the ability to reduce hydroperoxides in complex lipids (phospholipids, cholesterol, and cholesteryl esters), even when they are inserted into biomembranes or lipoproteins [38]. Thus, GPX4 is essential to prevent the accumulation of toxic lipid hydroperoxides, which can trigger a mode of cell death termed ferroptosis, associated with many cardiovascular diseases [39].

The profound effect exerted by the LANCL proteins on radicals-generating and -scavenging enzymes results in a significantly reduced ROS content in the overexpressing compared with the double-silenced H9c2 cells, particularly in mitochondria (Figure 4B). Interestingly, treatment with ABA of LANCL1/2-overexpressing cells further reduced mitochondrial ROS content (Figure 6B and Figure 8B), an effect which was not observed in double-silenced cells or in ERRα-silenced overexpressing cells. Thus, the beneficial effect of ABA in reducing cardiomyocyte mitochondrial ROS content requires a functional LANCL1/2–ERRα axis.

The results obtained here imply that the ABA/LANCL hormone/receptors system exerts a protective role on (mitochondrial) ROS production and that conversely a reduced activity of this signaling pathway necessarily results in an increased susceptibility of cardiomyocytes to oxidative stress. This conclusion may bear significant consequences in the clinical setting; on the one hand, the activation of the LANCL1/2-signaling pathway is attainable by means of pharmacological or nutraceutical compositions titrated in ABA [3]; on the other hand, reduced endogenous LANCL1/2 expression levels may be responsible for an increased susceptibility of cardiomyocytes to oxidative stress, a possibility worth exploring in patients with a poor outcome after cardiac reperfusion.

It should be emphasized that LANCL1/2-overexpressing cells have a significantly steeper proton gradient than double-silenced cells [6]; thus, it could be anticipated that they should also have a higher production of ROS as a consequence of a higher respiratory chain activity. Instead, double-silenced H9c2 cells, despite a much reduced ΔΨ compared with overexpressing cells, produce significantly more ROS. These profound differences in mitochondrial function and ROS generation between overexpressing and double-silenced H9c2 demonstrate the important role played by the LANCL1/2 proteins in the regulation of mitochondrial function. Moreover, the “improved” phenotype of LANCL1/2-overexpressing compared with double-silenced cells regarding ROS metabolism (lower transcriptional and protein levels of radicals-generating enzymes, higher levels of radicals-scavenging enzymes, and lower levels of cellular and mitochondrial ROS) is dependent on the transcription factor ERRα, as its knockdown abrogates all of the above features. A general, cell type-independent role of the LANCL1/2 receptor system in protecting cells against radicals also emerges from very recent reports on hepatocellular carcinoma cells and on testicular cells [40,41]. Specifically, a consistent negative correlation was observed between LANCL1 expression and ROS levels, as well as ROS-responsive gene expression, in hepatocellular carcinoma (HCC) cell lines [40]. Additionally, LANCL2 plays a crucial role in maintaining the redox balance in round spermatids, which is essential for acrosomal development and sperm quality. The overexpression of LANCL2 in NCCIT cells significantly improved cell viability when cells were treated with H2O2, and LANCL2 knockout testes showed reduced GSH and increased GSSG levels, decreased testicular SOD activity, and elevated levels of malondialdehyde (MDA) and protein carbonyl (PCO), indicating a disruption in testicular redox balance following LANCL2 deletion [41]. These findings indicate that LANCL1 and LANCL2 proteins play an important role in maintaining redox balance in cell types as different as hepatocytes, cardiomyocytes, and spermatids. 

Taken together with previously published results [4,6,25,26], the data reported here allow us to outline a multifaceted role of the ABA/LANCL1-2 hormone/receptors system in cardiomyocytes. Targeting this system, via LANCL1/2 overexpression or via stimulation with ABA of endogenous LANCL1-2 proteins (expression in the heart is the highest among non-neurological tissues [6]), results in the following responses: (i) increased NO production, both under normoxia and hypoxia, via eNOS activation by the AMPK/PGC-1α axis; (ii) increased mitochondrial respiration with higher basal and maximal respiration rates, a doubling of the spare respiratory capacity, and a steeper proton gradient (∆Ψ) under normoxia and after hypoxia/reoxygenation [6,25]; (iii) improved cell vitality after H/R; (iv) increased glucose uptake and oxidation, with a higher cell NADPH content; (v) increased fatty acid-fueled respiration rate [25]; (vi) increased O_2_ consumption under normoxia; (vii) reduced mitochondrial ROS content resulting from an improved enzymatic expression pattern, with less ROS-producing and more ROS-scavenging enzymes, in the face of increased mitochondrial electron chain transport activity. These pleiotropic functions controlled by the LANCL proteins in cardiomyocytes require a signaling pathway dependent on the activation of AMPK, PGC-1α, and ERRα, arguably among the most conserved and evolutionarily ancient nutritional signals [26], which increase glucose and fatty acid transport and oxidative metabolism, thus increasing cell energy production. This same signaling pathway also improves the cell capacity to withstand a higher ROS production, which occurs when oxygen becomes limiting with respect to the higher electron flow. The “backward” transfer of electrons at some of the respiratory complexes, resulting in the reduction of metabolites and coenzymes, can partly reduce electron “overflow”, at the expense of increased radicals generation [42,43,44,45,46,47,48,49,50,51]. Thus, a signaling pathway that increases mitochondrial energy production, as the LANCL/AMPK/PGC-1α/ERRα axis proves to achieve, also needs to increase the cell capacity to protect itself from oxidative stress, to ensure cell survival to the “feast”. Indeed, the same signaling axis was recently shown to “fine tune” electron flow along the respiratory chain by allowing the partial uncoupling of oxidative phosphorylation [25], thus reducing hyperpolarization and consequently retrograde electron flux [52,53].

## Figures and Tables

**Table 1:** Primer sequences used to amplify rat target genes.

Rat Genes	Accession N.	Forward Primer 5′-3′	Reverse Primer 5′-3′
Lancl1	NM_053723	TCTTGCTCCTCATCCTGCTCATC	CACTGTACTCGCCGAAGGTCTC
Lancl2	NM_001014187	GGTGCCACGGTGCTCCAG	CCTCGCTGCCAAATCACATCAC
Sod2	NM_017051	TAAGGGTGGTGGAGAACCCA	ACCTTGGACTCCCACAGACA
Nox4	NM_053524	CTGTACAACCAAGGGCCAGA	GCTCTGCTCAAACACAATCCT
Gpx4	NM_017165	CCGTCTGAGCCGCTTATTGA	AATCATCGCGGGATGCACA
Cox2	S67722	GTGAAAACTGTACTACGCCGAG	TACTGTGTTTGGGGTGGGCT
Xor	NM_017154	TCCCTGCGTTTGGTAGCATC	CCAGGAAAAGAGGTGGCTCC
Hprt1	NM_012583	TTGGTCAAGCAGTACAGCCC	TGGCCTGTATCCAACACTTCG

**Table 2:** Primary and secondary antibodies used for Western blot.

Primary Antibody	Host	Concentrations	Manufacturer
Anti-LANCL1	Rabbit	1:250	Novus Biologicals, Centennial, CO, USA
Anti-LANCL2	Mouse	1:1000	Reference [30]
Anti-XO	Mouse	1:100	Santa Cruz Biotechnology Inc., Santa Cruz, CA, USA
Anti-COX2	Goat	1:200	Santa Cruz Biotechnology Inc., Santa Cruz, CA, USA
Anti-SOD2	Rabbit	1:5000	Abcam
Anti-GPX4	Mouse	1:100	Santa Cruz Biotechnology Inc., Santa Cruz, CA, USA
Anti-ERRα	Mouse	1:200	Santa Cruz Biotechnology Inc., Santa Cruz, CA, USA
Anti-vinculin	Rabbit	1:1000	Cell Signaling Technology, Danvers, MA, USA
Anti-Actin	Mouse	1:1000	Santa Cruz Biotechnology Inc., Santa Cruz, CA, USA
**Secondary Antibody**	**Concentrations**		**Manufacturer**
Anti-Mouse	1:2000		Santa Cruz Biotechnology Inc., Santa Cruz, CA, USA
Anti-Rabbit	1:1000		Santa Cruz Biotechnology Inc., Santa Cruz, CA, USA
Anti-Goat	1:1000		Santa Cruz Biotechnology Inc., Santa Cruz, CA, USA

## Data Availability

The original contributions presented in the study are included in the article, further inquiries can be directed to the corresponding author/s.
